# Effects of pretesting implicit self-determined motivation on behavioral engagement: evidence for the mere measurement effect at the implicit level

**DOI:** 10.3389/fpsyg.2014.00125

**Published:** 2014-02-13

**Authors:** David A. Keatley, David D. Clarke, Eamonn Ferguson, Martin S. Hagger

**Affiliations:** ^1^Health Psychology and Behavioural Medicine Research Group, Laboratory of Self-Regulation, Faculty of Health Sciences, School of Psychology and Speech Curtin UniversityPerth, WA, Australia; ^2^Personality, Social Psychology, and Health Research Group, School of Psychology, University of NottinghamNottingham,UK

**Keywords:** implicit measurement, mere measurement effect, self-determination theory, implicit association test, priming

## Abstract

Research into individuals’ intended behavior and performance has traditionally adopted explicitly measured, self-report constructs, and outcomes. More recently, research has shown that completing explicit self-report measures of constructs may effect subsequent behavior, termed the “mere measurement” effect. The aim of the present experiment was to investigate whether implicit measures of motivation showed a similar mere measurement effect on subsequent behavior. It may be the case that measuring the implicit systems affects subsequent implicit interventions (e.g., priming), observable on subsequent behavior. Priming manipulations were also given to participants in order to investigate the interaction between measurement and priming of motivation. Initially, a 2 [implicit association test (IAT: present vs. absent) ×2 (Prime: autonomous vs. absent) and a 2 (IAT: present vs. absent) × 2 (Prime: controlled vs. absent)] between participants designs were conducted, these were them combined into a 2 (IAT: present vs. absent) ×3 (Prime: autonomous vs. controlled vs. absent) between participants design, with attempts at a novel task taken as the outcome measure. Implicit measure completion significantly decreased behavioral engagement. Priming autonomous motivation significantly facilitated, and controlled motivation significantly inhibited performance. Finally, there was a significant implicit measurement × priming interaction, such that priming autonomous motivation only improved performance in the absence of the implicit measure. Overall, this research provides an insight into the effects of implicit measurement and priming of motivation and the combined effect of completing both tasks on behavior.

## INTRODUCTION

When considering explicit, self-report assessments of motivation and other psychological constructs, recent research has shown that completion of a questionnaire significantly affects subsequent behavior, frequently referred to as the *mere measurement *effect (Godin et al., 2010; Conner et al., 2011). In order to better understand the full range of motivational antecedents of behavior, researchers have recently begun to incorporate implicit measures and processes into their models of behavioral prediction (e.g., Levesque et al., 2008; Banting et al., 2009). In particular, [self-determination theory (SDT; Deci and Ryan, 2008)] has been augmented to include implicitly measured motivational variables alongside explicit factors. While a mere measurement effect has been observed for explicit questionnaire measures, an important outstanding question is whether the mere measurement effect generalizes to implicit measures. In the current study, we adopted an experimental design to investigate this possibility. The aim and unique contribution to the literature of the present research, therefore, was to investigate the effects of implicitly measured motivational factors. In addition, we investigated whether mere measurement affected or sensitised subsequent priming manipulations on behavioral engagement, within the framework SDT (Deci and Ryan, 1985).

### THE MERE MEASUREMENT EFFECT

Responding to an explicit questionnaire can affect an inidividuals’ subsequent behavior (Fitzsimons and Morwitz, 1996; Morwitz and Fitzsimons, 2004). These effects of measurement on evaluations and behavior has been termed the *mere measurement effect* (Godin et al., 2010; Conner et al., 2011). One explanation for why the mere measurement occurs is that measuring intentions increases the saliency or accessibility of thoughts related to the outcome behavior; therefore, subsequent behavior is more consistent with the thoughts that have been made more accessible or salient as a result of the measures (Morwitz et al., 1993). Powell and Fazio (1984) and Fazio et al. (1986) provided an account for this in terms of self-report completion activating nodes for the outcome behavior in question. Essentially, questioning a respondent about their intentions to perform a particular activity (e.g., intention to eat fruit and vegetables) leads to spreading activation of related category nodes (e.g., health beliefs, dieting, weight loss etc.).

Research into the mere measurement effect has solely focused on explicit, self-report measures. Recently, studies focusing on the prediction of behavior have begun to incorporate implicit measures of motivation (e.g., Keatley et al., 2012, 2013). Implicit measures, such as the implicit association test (IAT; Greenwald et al., 1998) assess the strength of underlying associations between target (e.g., motivation) and attribute (e.g., self) categories. Measurement of these underlying associations may result in a similar spreading activation, and accessibility processes that are outlined as explanations for the mere measurement effect. Within the realm of implicit process this would be akin to suggesting that the measurement of an implicit cognition would influence subsequent behavior. There is also growing evidence that measuring an individual’s motivation may influence how well the person responds to an intervention (Solomon, 1949; Braver and Braver, 1988; McCambridge et al., 2011). It may well, therefore, be the cases that measuring the implicit system will also sensitize that person to a subsequent implicit intervention (e.g., priming) and this would be observed on subsequent behavior.

### IMPLICIT MOTIVATION AND MERE MEASUREMENT

Self-determination theory has been applied to a range of behaviors (Edmunds et al., 2007; Deci and Ryan, 2008), typically using explicit measures (Biddle et al., 1999; Chatzisarantis et al., 2003; Hagger et al., 2003; Banting et al., 2011). Within SDT different forms of motivation are proposed to provide an account of differences in observable performance. When individuals are *autonomously* motivated, they will feel as sense of authorship or choice for engaging in an activity; therefore, they are more likely to enjoy that behavior and persist without the need for external pressure. In contrast, individuals may perform a behavior due to external sources of pressure or control, leading to *controlled* forms of motivation. If an individual feels controlled, they are likely to persist at a behavior only as long as the external reinforcement is salient (Deci et al., 1999). In the current study, individuals’ implicit motivation was measured using an IAT for autonomous motivation orientation relative to controlled motivation. The behavioral outcome was as the number of attempts made on a novel problem-solving task (see Baumeister et al., 1998; Moller et al., 2006). An implicit mere measurement effect would be indicated if simply competing an IAT alters the behavioral response. Participants’ autonomy was not at risk of being undermined as the problem-solving task was presented in a free-choice paradigm wherein participants could stop at any time (Hagger and Chatzisarantis, 2011).

A further area of investigation, in addition to implicit mere measurement effects, was whether completion of implicit measures would interact with an intervention (e.g., priming motivation) to change subsequent behavior. Several studies have investigated the effect of priming of motivational constructs from SDT on behavior (Levesque and Pelletier, 2003; Ratelle et al., 2005; Burton et al., 2006). The most notable of these, in relation to the current research, was the research conducted by Levesque and Pelletier’s (2003). In the first of a series of experiments, a scrambled sentence task (SST; Srull and Wyer, 1979) was used, which comprised words inherently linked with either autonomous or controlled motivation. Priming significantly altered performance on challenging puzzles to match those with similar chronic motivation orientations. A further study investigated whether chronically accessible motivational orientations would moderate the effects of priming constructs linked to motivation (Deci and Ryan, 1985. In their study a free-choice paradigm) was used as a measure of participants’ motivation to persist with a task when the experiment had ostensibly finished. Results indicated differential effects of priming, such that only those without chronically accessible motivation orientations exhibited changes in behavior after priming (Deci and Ryan, 1985). Levesque and Pelletier’s research provides an important contribution to the inception of the current research in terms of the effects of priming motivation. However, while they focused on explicitly measured motivation orientations, the present research provides an important advancement in incorporating an implicit measure of motivation.

### THE PRESENT STUDY

The aim of the present study was to test for the possibility of a mere measurement effect with implicit measures of motivation from SDT. In the current study, it was hypothesized that a mere measurement effect would be observed at the implicit level such that measuring implicit motivation would lead to subsequent changes in behavior. Thus the first hypothesis (H1) was that completion of an implicit measure of motivation is sufficient to alter peoples behavioral responses. A second hypothesis (H2) was that a priming manipulation would significantly affect subsequent motivation in the direction of the priming, independent of the implicit measurement. In the absence of any previous results or indeed any strong theoretical prediction, a final more exploratory aim of this experiment was to examine the interaction between prime type (autonomous vs. controlled vs. none) and implicit measure (present vs. absent). This is tested with a two-tailed significance.

## MATERIALS AND METHODS

### DESIGN

Originally, two between subjects experiments experiments were conducted: (1) A 2 (Implicit measure: present vs. absent) by 2 (priming: autonomouos vs. absent) and (2) a separate 2 (Implicit measure: present vs. absent) by 2 (priming controlled vs. absent) experiment. These were them combined into a 2 (Implicit measure: present vs. absent) by 3 (priming: autonomous vs. controlled vs. absent) between-participants design for ease of comparison of effects. Attempts at completing a novel problem-solving task was the dependent variable.

### PARTICIPANTS

Undergraduate students (*N* = 80; 52 female, 28 male, *M* age = 20.50, age range: 19–46) from the University of [name and country omitted for masked review] participated in the study (See **Table 1**). Students were contacted via emails detailing the study and the opportunity to participate. A $6 inconvenience allowance was provided for participation. The ethics committee in the School of Psychology at the University of [omitted for masked review] approved the study protocol. We combined data from two studies that were conducted separately, one focusing on primes for autonomous motivation and one focusing on primes for controlled motivation. In each separate experiment participants were randomally allocated to conditions. There were no demographic differences between experiment groups and therefore they were combined for parsimony.

**Table 1:** Descriptive statistics and participants in each group and correlations between IAT-completing groups, and number of attempts made.

Group	IAT	Prime	*n*	Attempts mademean (*SD*)	Correlation
1	×	Autonomy	10	20.40 (6.72)	-0.25
2	×	Control	10	13.70 (6.36)	-0.13
3	×		20	16.70 (8.98)	-0.22
4		Autonomy	10	33.10 (9.13)	-
5		Control	10	12.10 (5.38)	-
6			20	17.90 (5.02)	-

*X in the column indicates IAT was present. Correlation is between IAT-D scores and attempts made for that group.*

### MEASURES AND EXPERIMENTAL MANIPULATIONS

#### Implicit association test

Implicit measurement of autonomous and controlled motivation was measured using a modified IAT. The underlying principle of the IAT is that the presentation of paired category and attribute stimuli that are strongly associated in memory (e.g., words like *self* and *autonomous*) will result in faster response latencies compared to paired category and attribute stimuli that are weakly associated (e.g., words like *self* and *controlled*). Words representing autonomous (*choice, free, spontaneous, willing, authentic*) and controlled (*pressured, restricted, forced, should, controlled*) motivation and words pertaining to “self” (*I, me, my, mine, self*) and “others” (*others, they, them, their, theirs*) were taken from research conducted by Levesque and Brown (2007), in which they were shown to offer distinct representations. Further information was also provided explaining the differences between the motivation types. The category “others” was fully explained and introduced as being “not-self,” rather than a more social-comparison category, and previous research has also incorporated these labels (e.g., Brunstein and Schmitt, 2004). A standard five-step IAT was used. Blocks one, two, and four were for practice, each consisting of 20 trials; test blocks (three and five) comprised 60 trials – 20 practice and 40 test. The IAT effect was calculated using the improved *D*-score algorithm (Greenwald et al., 2003). Coding was such that higher scores were indicative of an autonomous motivation orientation relative to controlled motivation.

#### Priming of autonomous motivation

Autonomous motivation was primed using a SST. Participants were presented with a series of 15 sentences in which the word order was scrambled. Participants were instructed to use four of the five words in each scrambled sentence to create a grammatically correct sentence. Based on previous findings (Srull and Wyer, 1979; Levesque and Pelletier, 2003), prime words were incorporated into 12 of the items (80%). Prime words were: *spontaneous*, *challenge*, *interested*, *volunteered*, *involved*, *satisfied*, *autonomous*, *mastering*, *delighted*, *absorbed*, *competent*, and *enjoying*. An example of the type of scrambled sentence is: “has challenge he a chair.” Participants could create two grammatically correct sentences; one that included the prime word and another that did not.

#### Priming of controlled motivation

 For the priming controlled motivation condition, the key prime words included in the SST were: *competitive*, *obligation*, *expected*, *evaluated*, *constrained*, *demanded*, *avoiding*, *restricted*, *forced*, *pressured*, *controlled*, and *proving*. The words were again embedded in the scrambled sentences (e.g., “is quiet competitive very she”). The sentences were the same as the autonomous priming condition, the only exception was that the autonomous motivation stimulus words were substituted for the controlled motivation words.

#### Outcome variable

 Number of attempts on the figure-tracing task was the dependent variable as this allows for close comparison with Levesque and Pelletier (2003) research that focused on number of solutions provided for a crossword puzzle. The tracing tasks were the same as those used by Baumeister et al. (1998). Participants were unaware they were impossible to complete. This was not specifically assessed; however, feedback from the majority of participants indicated that none were aware. The task has two practice figures that were possible to complete (all participants completed them), and two test figures, that were impossible to complete. The outcome variable was the combined attempts at both tasks.

#### Test of awareness

As outlined in Chartrand and Bargh (1996), participants’ awareness of the nature of the primes that they were exposed to was measured. At the end of the study, participants were asked (a) whether they had done the separate parts of the study as unrelated tasks and (b) whether anything they had done in the first sections affected what they had done in the experimental task (item recoded). These were answered on a 7-point likert-type scale (1 = *do not agree at all*, 7 = *agree completely*).

### PROCEDURE

Participants were invited into the laboratory and tested individually. Participants received sufficient information for each section of the study and signed an informed consent form prior to the commencement of data collection. Participants were randomly assigned within each of the 2×2 designs. Depending on allocation, participants completed either: an IAT and an autonomy-related SST; an IAT and a control-related SST; the IAT alone; SST alone; or no pre-test measure or prime.

For the behavioral measures participants were instructed by the researcher to complete the figure-tracing task according to a protocol provided by Moller et al. (2006). Participants were required to trace several geometric figures without taking their pencil from the page once they started, and without retracing any line once drawn. Participants were initially given two solvable figures to trace, in order to confirm they understood the rules and process. Multiple slips of paper were provided so that participants could make as many attempts as they wanted. After completion of the practice trials, the researcher administered the test figures. Participants were unaware that the test figures were unsolvable. The researcher then left the room, for the participants to attempt the task for as long as they wanted. A maximum of 20 min was set, any participants still working after this time were told to stop.

## RESULTS AND DISCUSSION

### PRELIMINARY ANALYSIS

Awareness checks indicated that participants viewed the separate parts of the study as unrelated (*M* = 4.83, *SD*= 0.83), and that completion of the experimental task was not affected by what they had previously done (*M* = 6.04, *SD*= 0.82). All participants in the scrambled sentence conditions reported at least four (on a seven-point scale) on both awareness check questions. Correlation coefficients between awareness checks and experimental task were non-significant (*p*s > 0.30). No participants reported any suspicion or awareness of the priming manipulation.

### EFFECTS OF IAT AND PRIMED MOTIVATION

Data were initially pooled from the two separate 2×2 designs^1^. The difference in attempts made between the groups was examined with a 2 (IAT measure: present vs. absent) × 3 (Priming: autonomous motivation vs. controlled motivation vs. no priming) analysis of variance (ANOVA) – see Table 1 ^2^. The ANOVA revealed a significant main effect for the IAT, *F*(1,74) = 5.90, *p* = 0.02,$\eta_{p}^{2}$ = 0.07, such that participants completing the IAT made fewer attempts on the figure-tracing task (*M* = 16.88, *SD* = 8.05) than those that did not complete the IAT (*M* = 20.25, *SD* = 10.02). This is indicative of a “mere measurement” effect.

A significant main effect of priming motivation was also found, *F*(2,74) = 19.96, *p* < 0.001,$\eta_{p}^{2}$ = 0.35, such that participants that received the prime for autonomous motivation made more attempts (*M* = 26.75, *SD* = 10.12) than those that received the prime for controlled motivation (*M* = 12.90, *SD* = 5.79) and those that did not receive any priming manipulation (*M* = 17.30, *SD* = 7.21).

**FIGURE 1:**
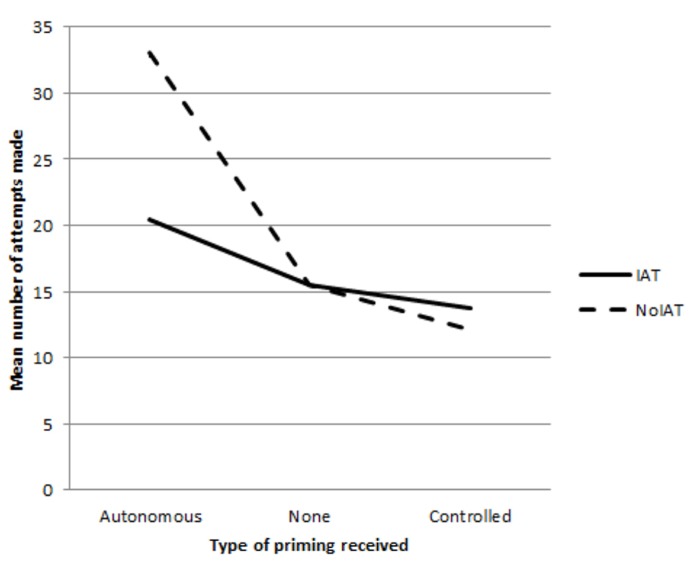
** Graph showing the interaction of IAT completion and priming manipulation on number of attempts on a figure-tracing task**.

Finally, a significant IAT completion x priming condition interaction effect was found, *F*(2,74) = 6.40, *p* = 0.01,$\eta_{p}^{2}$ = 0.15 (see **Figure 1**). Analysis of simple effects revealed a significant difference in number of attempts in the no-IAT condition only for those who received the prime for autonomous motivation (*M* = 33.10, *SD* = 9.13) compared to those who did not receive the prime (*M* = 17.90, *SD* = 5.02), *F*(1,56) = 36.03 *p* < 0.001,$\eta_{p}^{2}$ = 0.39. There was also a significant difference in attempts within the prime for autonomous motivation condition for those who did not receive the IAT (*M* = 33.10, *SD* = 9.13) and those that did (*M* = 20.40, *SD* = 6.72), *F*(1,56) = 14.15, *p* < 0.001,$\eta_{p}^{2}$ = 0.20. Furthermore, there was a significant difference in number of attempts made in the no-IAT condition for those who received a prime for autonomous motivation (*M* = 33.10, *SD* = 9.13) compared to those who received a prime for controlled motivation (*M* = 12.10, *SD* = 5.38), *F*(1,36) = 44.54, *p* < 0.001,$\eta_{p}^{2}$ = 0.55. There was a significant difference when the IAT was administered between those who then received a prime for autonomous motivation (*M* = 20.40, *SD* = 6.72) and those who then received a prime for controlled motivation (*M* = 13.70, *SD* = 6.36), *F*(1,36) = 16.29, *p* < 0.001,$\eta_{p}^{2}$ = 0.31.

## GENERAL DISCUSSION

The aim of the present investigation was to investigate whether completion of an implicit measure would alter behavioral responses. Although there is an expanding literature that demonstrates that completion of explicit measures of motivation or other psychosocial constructs has a significant effect on behavior (Godin et al., 2010; Conner et al., 2011), research in the implicit domain has seldom systematically investigated this phenomenon. The present study is the first to test this in the field of SDT and motivation, and seems to indicate there is potential for the completion of implicit measures to affect behavior (H1). We further hypothesized (H2) that priming autonomous motivation would significantly increase performance, and that priming controlled motivation would significantly decrease performance, in terms of the number of attempts made on the figure-tracing task. This was supported, and provides further support for the effectiveness of priming on behavioral engagement (Levesque and Pelletier, 2003; Ratelle et al., 2005; Burton et al., 2006).

The final aim was to investigate the interaction between implicit measure of motivation from SDT and the prime used to activate these motivational orientations. An interaction was found and indicated that the prime of autonomous motivation led to increased attempts at a novel problem-solving task only when participants did not complete the implicit measure of autonomous motivation. This is very important in relation to future research adopting implicit measures of motivation from SDT, especially if this is followed by a behavioral outcome task. To speculate, it is possible that the relative implicit measure of motivation may sensitize or interfere with individuals’ motivation to affect processing, regardless of how the IAT is scored. There is evidence to suggest that IAT measures of autonomous and controlled motivation, when reduced to a relative measure, may be more akin to a generalized measure of controlled, rather than autonomous, motivation (Harris, 2008). The IAT measure may therefore have had the effect of priming controlled motivation, and inhibited the effectiveness of the subsequent prime of autonomous motivation. Future research could use “known groups” of participants (i.e., groups for which the implicit motivation orientation is already known) and measure whether the interaction of further implicit measurement, and priming maintains this effect, or whether their motivation orientation interacts with the implicit measurement. Essentially, those with a particular motivation orientation may have this orientation facilitated through further measurement, which then interacts with priming or manipulation procedures. Further research is needed to investigate this possibility, and whether the fatigue effect is reduced by brief versions of the IAT (e.g., Sriram and Greenwald, 2009). A further area of research is whether implicit measures might actually mediate priming on performance. A follow-up study could switch the order of priming and implicit measure of motivation in the Solomon four-group design such that the effects of priming on performance with or without implicit measurement could be investigated.

In terms of SDT, the current findings continue a growing trend in research indicating the important role of implicit processes on behavior (Conner et al., 2007; Ahern et al., 2008). The current study adds support to the effects of priming motivation (Levesque and Pelletier, 2003); and adds to the literature by highlighting a connection between implicit measures of motivation, and priming of motivation. The current findings add to previous research that has augmented SDT with implicit measures of motivation (Keatley et al., 2012). Future research investigating key tenets of SDT could adopt single-category implicit measures (e.g., the single-category IAT, Karpinski and Steinman, 2006) as SDT proposes individuals may have both autonomous and controlled motivation orientations, rather than a dichotonomous measure representing autonomous vs. controlled motivation.

It is important for future research to investigate the effects of different priming manipulations on behavioral engagement, to assess whether the mere measurement effect occurs for explicit/supraliminal primes, and implicit/subliminal. Given the implicit nature of measures such as the IAT, there may be greater effects on subsequent behavioral engagement following manipulation of the impulsive system via implicit/subliminal primes..

The current research is important in providing further support for the effects of motivation orientations, as proposed by SDT, at an implicit level on behavioral engagement. While there are several studies into the predictive validity of implicit measures of motivation, and the effects of priming of motivation, the present studies are distinctive in bringing together these two paradigms to investigate their combined effects on behavior. Attention should be given to the effect of completing implicit measures of motivational orientations from SDT on behavior, above-and-beyond the outcomes of the IAT itself. Furthermore, attention should be given to the interaction between measuring implicit motivation, as proposed by SDT, and processes that could prime these motivational orientations, in future studies.

## Conflict of Interest Statement

The authors declare that the research was conducted in the absence of any commercial or financial relationships that could be construed as a potential conflict of interest.

## Footnotes

1 No significant difference was found between mean IAT-D scores for participants in each experiment who completed the IAT, *F* (2,37) = 1.07, *p* = 0.35, $\eta_{p}^{2}$ = 0.06.

2 Correlations between IAT-D scores and attempts made for groups were also calculated. There were no significant correlations between IAT-D scores and attemtps made. There were also no significant differences in IAT-D scores between groups, *F* (2,37) = 1.07, *p* = 0.35.
